# Focal task specific dystonia: a review and update

**DOI:** 10.1007/s00415-016-8373-z

**Published:** 2016-12-30

**Authors:** Christine M. Stahl, Steven J. Frucht

**Affiliations:** Icahn School of Medicine at Mount Sinai, 5 East 98th Street, Box 1138, First Floor, New York, NY 10029 USA

**Keywords:** Focal task specific dystonia (FTSD), Writer's cramp, Musician's dystonia

## Abstract

In this review, we summarize recent advances in understanding the etiology, risk factors and pathophysiology of focal task specific dystonia (FTSD), movement disorders characterized by abnormal motor activation during the performance of specific, repetitive actions. We focus on two common FTSD, musician’s dystonia and writer’s cramp. FTSD may pose a threat to the patient’s livelihood, and improved therapeutic treatments are needed.

## Introduction

Dystonias are a diverse “group of movement disorders characterized by sustained or intermittent muscle contractions causing abnormal, often repetitive, movements, postures, or both” [1]. Recently updated consensus opinion classifies dystonias by two axes: clinical characteristics and etiology. The first axis, clinical characteristics, includes the age of onset, affected body region, temporal pattern, and associated neurologic or systemic features [1]. A subclassification includes focal task specific dystonias (FTSD), which are a diverse group of focal dystonias affecting an isolated body part and are triggered, at least initially, by a specific action.

While the term “dystonia” was first used in 1911 by Oppenheim, the clinical phenomenon had been described almost a century earlier in patients with FTSD [2]. In 1830, clerks in the British Civil Service were noted to develop difficulty with writing. After observing these clerks, Sir Charles Bell remarked that he “found the action necessary for writing gone, or the motions so irregular as to make the letters be written zig-zag, whilst the power of strongly moving the arm for fencing remained…” [3]. Later in the 1860s, Samuel Solly labeled this condition “scrivener’s palsy” [4]. While “scrivener’s palsy” or writer’s cramp, as it is now called, is one of the more recognized forms of FTSD, dystonia may affect musicians, typists, hairdressers, painters, shoemakers and tailors [5–7]. Sport-related FTSD have also been described in golfers, pistol shooters and ping-pong players, among others. Because of their association with repetitive, fine motor tasks often linked to one’s profession, FTSD have also been referred to as occupational dystonias. Interestingly, until the 1980s FTSD were erroneously interpreted to be psychogenic in origin, often termed “occupational neuroses.” In 1982, evidence for an organic etiology emerged from the work of Sheehy and Marsden [8].

In this review, we discuss the phenomenology and epidemiology of two of the most common forms of FTSD, writer’s cramp and musician’s dystonia. We then discuss emerging findings on the etiology, pathophysiology and treatment of FTSD.

## Background

FTSD typically begins in adulthood with symptom onset in the third to sixth decade. Unlike other adult onset primary focal dystonias, FTSD is more common in men, and it usually affects the arm, facial muscles or larynx [9]. Overall prevalence estimates for FTSD in the general population range from 7 to 69 per million [10, 11]. However, prevalence has been estimated to be much higher in selected groups; for instance, some studies have shown as many as 14% of patients seen at performing arts medical centers have FTSD [12, 13].

FTSD typically presents as an insidious, painless loss of dexterity triggered by performance of a specific, often over-practiced task. Symptoms progress over time to trigger uncontrolled activation of muscle groups, leading to abnormal postures and movements. Early in the disease course, the dystonia typically is triggered only by the performance of a specific task, but over time spreads to involve other tasks, or even spreads to previously unaffected areas of the body. As with other types of dystonias, sensory tricks, or *geste antagonistes*, may temporarily reduce the dystonic symptoms of FTSD [14].

## Writer’s cramp and musician’s dystonia

Writer’s cramp is characterized by involuntary cramping of muscles of the hand, forearm, or upper arm selectively triggered by writing. Typically, the distal muscles of the upper extremity are affected, but dystonia may progress to include more proximal muscle groups, may be triggered by other activities, and can even spread to the opposite non-dominant hand. Average age of onset is in the fourth decade, and once present, symptoms rarely remit [8].

FTSD seen in musicians, a group that may be at risk due to overly practiced fine motor tasks, typically manifests in two phenotypes based on the particular instrument: musician’s hand dystonia or embouchure dystonia. Musician’s dystonia can occur in both amateur and professional musicians, men are affected four times as often as women, and average symptom onset is in the fourth decade [15].

Musician’s hand dystonia has been reported with a variety of instruments, including piano, violin, guitar, flute, clarinet, horn and tabla, among others. Dystonia typically occurs in the hand that performs the more demanding tasks, such as the right hand in pianists and the left hand in violinists [16]. The specific pattern of abnormal muscle activation varies by instrument. For example, abnormal flexion of the fingers is typically seen in pianists and violinists, while in woodwind or brass players, extension due to lumbrical activation can occur [15]. FTSD may be exquisitely task specific, triggered by playing one instrument, but sparing the hand when a patient plays a different instrument.

Embouchure dystonia may affect brass and woodwind players, with age of onset in the fourth decade [6]. The embouchure is the critical interplay of the lips and facial muscles with the instrument’s mouthpiece that controls the production of the desired air stream. Embouchure dystonias may be further classified by the pattern of abnormal movements, including embouchure tremor, involuntary lip movements and jaw closure [6].

## Advances in understanding

In recent years, much effort has focused on understanding the etiology, risk factors and pathophysiology of FTSD and potential therapeutic interventions. We will review these in turn.

### Etiology

The etiology of FTSD remains unknown, although recent lines of evidence suggest that both genetic and environmental factors are important [17]. Examination of family members of patients with FTSD revealed up to 25% of patients with an affected family member [18, 19]. This is consistent with a recent study of musician’s dystonia which found approximately 20% of patients with a similarly affected family member [20]. A recent genome-wide analysis has found an association with the arylsulfatase G (ARSG) gene in both musician’s hand dystonia and writer’s cramp, but to date, a specific causative mutation within this gene has not been identified [21, 22]. Additionally, in a study of musicians with FTSD of the hand, along with patients with writer’s cramp and their relatives, reduced interhemispheric inhibition as measured by transcranial magnetic stimulation (TMS) was observed in individuals where there was a positive family history for dystonia. This finding suggests that reduced interhemispheric inhibition may serve as a possible endophenotypic marker of genetic susceptibility for developing FTSD [23].

In addition to repetitive, over-practicing of a motor task, other environmental factors may contribute to the risk factors of developing FTSD. Possible risk factors include personality traits, such as perfectionism and anxiety, anatomical factors, such as hand size and joint mobility, as well as delayed onset of age of musical training [17, 24, 25].

### Pathophysiology

The pathophysiology of FTSD has been linked to abnormalities in inhibition, plasticity, and motor networks. In 1995, experiments first demonstrated decreased short intracortical inhibition (SICI) in FTSD patients compared to healthy controls using TMS [26]. Interestingly, this abnormality was found in the bilateral hemispheres of patients, despite unilateral symptoms. Recent research has therefore postulated that decreased SICI may not directly cause abnormal motor activation but rather facilitate the development of FTSD through other mechanisms [27, 28]. Specifically, one suggested mechanism, found in several studies of FTSD patients, is the development of impaired surround inhibition, a neural inhibitory mechanism responsible for the selective recruitment and activation of muscles necessary for a particular task with inactivation of the neighboring muscles that are unnecessary [27, 29, 30]. Consistent with the hypothesis of decreased SICI and impaired surround inhibition, multiple studies have shown a loss of dexterity and impaired independent movement of fingers of patients with either writer’s cramp or musician’s dystonia [31–33].

Another interesting abnormality that may contribute to FTSD pathology is maladaptive neural plasticity. While plasticity is believed to be critical to the processes of learning and memory, maladaptive neuroplastic responses in both the motor and sensory cortices have been examined in conditioning protocols using repetitive stimuli from TMS [34]. Patients with writer’s cramp exhibited abnormal responses to paired associative stimulation of the median nerve and primary motor cortex. Such abnormalities included increased facilitation with spread to non-median nerve innervated muscles in addition to the absence of a typical cortical silent period [35]. More recently, experiments demonstrated a decreased short latency afferent inhibition following 1-Hz repetitive TMS in writer’s cramp, but not in normal controls [36].

Furthermore, in both musician’s dystonia and writer’s cramp, functional neuroimaging experiments have demonstrated abnormal cortical representations of digits and reorganization of the sensory homunculus in the sensory cortex [37–39]. Such aberrant somatotopy may be reversible with associated improved fine motor control using constraint-induced therapy [40–42]. However, another study of somatotopic mapping discovered bilateral misrepresentation of digits despite unilateral dystonic symptoms, suggesting that the disturbed somatotopy is an endophenotype for vulnerability to develop FTSD [43]. Regardless of the etiology of the aberrant somatotopy, the evidence suggests that maladaptive plasticity of FTSD impairs sensorimotor integration.

Finally, results from recent investigations have suggested a network disorder leading to FTSD, whereby involvement of the entire sensorimotor network contributes to dystonia [44]. Hyperactivation of the basal ganglia has been demonstrated in fMRI studies of writer’s cramp [45]. Further aberrant basal ganglia function has been demonstrated by PET studies, which have shown decreased release of striatal dopamine during hand activation in patients with writer’s cramp [46]. Cerebellar dysfunction has also been suggested to contribute to FTSD, although the specific abnormality is not well understood. Some investigations have reported increased cerebellar activity in patients with writer’s cramp [47–49], while others have demonstrated decreased activity in the cerebellum during hand activation in FTSD [50, 51]. Further research is warranted to understand the precise network abnormalities; however, evidence of aberrant connections between the basal ganglia and cerebellum leading to dystonia is supported by research demonstrating that interruption of this connection leads to improvement of the dystonic symptoms [52].

### Treatment

Current treatment modalities for FTSD include oral medication, chemodenervation, surgery and physical therapy. Anticholinergic agents like trihexyphenidyl, as well as other medications, such as primidone, baclofen, and phenytoin have been tried with inconsistent responses and frequent intolerable side effects [53–55]. Chemodenervation with botulinum neurotoxin (BoNT) type A has been the mainstay of treatment for FTSD.

Each of the seven known BoNT serotypes (types A–G) targets a specific SNARE protein for degradation in peripheral cholinergic neurons, thereby preventing the downstream release of acetylcholine into the neuromuscular junction. As a result, chemodenervation and subsequent muscle paralysis occur and persist for several months until the eventual degradation of BoNT and regeneration of SNARE proteins [56]. While the inhibition of acetylcholine release at the neuromuscular junction is believed to be a major component of BoNT’s mechanism of action, there is increasing evidence that BoNT also acts peripherally at gamma motor neurons to reduce afferent sensory input from muscle spindles to the central nervous system and to alter sensorimotor pathways [57–61]. Treatment of limb dystonia with BoNT has demonstrated transient increased intracortical inhibition on par with normal levels of inhibition as measured by transcranial magnetic stimulation [62]. Furthermore, recent research suggests that BoNT may additionally have non-SNARE cellular targets involved in wide-ranging activities, such as cell division and apoptosis, neuritogenesis and gene expression [63].

Of the seven BoNT serotypes, only serotype A and to a lesser extent serotype B are available for clinical use, with specific formulations of each serotype characterized by different potency, immunogenicity, preparation, compound stability and heat tolerance. Notably, BoNT type B is only formally approved for the treatment of cervical dystonia, while BoNT type A has approved indications in the treatment of both neurologic and non-neurologic conditions [64]. Multiple studies have demonstrated long-lasting treatment benefits of BoNT in FTSD, but there is a delicate balance between reducing dystonic symptoms without inducing concurrent residual weakness resulting in loss of motor function [53, 65–68]. Even with treatment, many affected musicians are no longer able to play professionally, due to the high level of fine motor skill required for continued professional performance.

In recent years, emerging studies have investigated the role of surgery and sensorimotor retraining as therapeutic options. Thalamotomies have been performed as treatment of a variety of movement disorders since the 1950s [69]. In what was the largest published case series of patients with writer’s cramp undergoing stereotactic ventro-oral thalamotomy, eleven of twelve patients reported almost complete resolution of symptoms with sustained benefit for over one year after surgery [70]. Based on its benefit for writer’s cramp, stereotactic ventro-oral thalamotomy was demonstrated to improve medically refractory musician’s dystonia with long-term benefit [71]. More recently in 2016, treatment with noninvasive gamma knife ventro-oral thalamotomy was shown to be effective in a case of refractory musician’s dystonia for a patient who was deemed too high-risk for conventional stereotactic thalamotomy [72]. However, larger long-term follow-up studies will be necessary to evaluate the lasting efficacy of this intervention. Additionally, a small case series has investigated the role of deep brain stimulation (DBS) in the treatment of FTSD with promising results [73]. Given the invasive nature of both thalamotomies and DBS, these procedures have primarily been reserved for medically refractory cases.

Based on the idea of excessive motor excitability and aberrant sensorimotor integration in the development of FTSD, sensorimotor retraining may hold promise. Previous attempts at reducing focal dystonia symptoms by means of rehabilitation involved immobilization and splinting of the affected body part. Recent studies examining the effects of augmenting current rehabilitation techniques to include transcranial direct current stimulation have offered encouraging results. In 2014, patients with musician’s dystonia displayed improvement of fine motor movements following motor retraining assisted by bi-hemispheric, noninvasive brain stimulation via transcranial direct current stimulation to the motor cortex [74]. Likewise, in 2015, transcranial direct simulation was shown to enhance the response to rehabilitation in patients with FTSD of the hand in a randomized control trial [75]. Anodal transcranial direct current stimulation targeting the cerebellum has also been shown to improve handwriting in patients with writer’s cramp [76].

## Conclusion

FTSD are a fascinating group of movement disorders characterized by aberrant motor overactivation during the performance of a specific, often over-practiced activity. The triggering activity can be associated with one’s occupation, leading to the disorder’s further classification as an occupational dystonia. The development of such a condition can impact one’s livelihood, particularly if symptoms are severe. While progress has been made in recent years in understanding the etiology, risk factors and pathophysiology of FTSD, improved therapeutic options are needed.
